# Beta-Carotene Affects the Effects of Heme Oxygenase-1 in Isolated, Ischemic/Reperfused Rat Hearts: Potential Role of the Iron

**DOI:** 10.3390/molecules27093039

**Published:** 2022-05-09

**Authors:** Evelin Csepanyi, Alexandra Gyongyosi, Istvan Lekli, Arpad Tosaki, Istvan Bak

**Affiliations:** Department of Pharmacology, Faculty of Pharmacy, University of Debrecen, 4032 Debrecen, Hungary; csepanyi.evelin@pharm.unideb.hu (E.C.); gyongyosi.alexandra@pharm.unideb.hu (A.G.); lekli.istvan@pharm.unideb.hu (I.L.); tosaki.arpad@pharm.unideb.hu (A.T.)

**Keywords:** oxidative stress, beta-carotene, heart, ischemia/reperfusion, heme-oxygenase-1, iron, desferrioxiamine

## Abstract

Beta-carotene (BC) is a well-known antioxidant. However, increasing evidence shows that under severe oxidative conditions, BC can become pro-oxidant, an effect that may be enhanced in the presence of iron (II). In our earlier studies, we observed that despite increasing heme oxygenase-1 (HO-1) levels in the heart, the protective effects of BC have been lost when it was used at a high concentration. Since iron releases from heme as a consequence of HO-1 activity, we hypothesized that the application of an iron-chelator (IC) would reverse the lost cardiac protection associated with an elevated HO-1 level. Thus, in the present study, we investigated the effects of desferrioxiamine (DFO) in isolated, ischemic/reperfused rat hearts after long-term treatment with vehicle or high-dose (HD) BC. Vehicle or 150 mg/bw kg daily doses of BC were administered to the rats for 4 weeks, and then their hearts were removed and subjected to 30 min of global ischemia (ISA) followed by 120 min of reperfusion (REP). During the experiments, cardiac function was registered, and at the end of the REP period, infarct size (IS) and HO-1 expression were measured. The results show that DFO treatment alone during REP significantly ameliorated postischemic cardiac function and decreased IS, although HO-1 expression was not increased significantly. In hearts isolated from BC-treated rats, no cardioprotective effects, despite an elevated HO-1 level, were observed, while DFO administration after ISA resulted in a mild improvement in heart function and IS. Our results suggest that iron could have a role whether BC exerts antioxidant or pro-oxidant effects in ISA/REP-injured hearts.

## 1. Introduction

Despite intensive research worldwide, there is a constant increase in the number of patients suffering from any kind of oxidative stress- (OS) related disease [1]. The imbalanced redox homeostasis of the cells during OS initiates further pathologic processes, finally leading to the damage of biological macromolecules, including DNA, proteins and membrane lipids, resulting in cell death [2]. Persistent oxidation of these molecules further exacerbates the evolved OS and facilitates the development and/or progression of several diseases, including cancer [3], diabetes [4], neurodegenerative diseases [5,6], atherosclerosis [7] or cardiovascular disorders [8,9,10]. Furthermore, OS also affects animal function, as well as meat or milk production [11,12], which consequently could have an impact on human nutrition and health.

The detrimental effects of OS can be reduced by the administration of exogenous antioxidants. The most favored exogenous antioxidants are flavonoids, ascorbic acid, vitamin E and carotenoids, including BC. Although BC has well-documented antioxidant properties and health effects [13], a number of studies highlight the pro-oxidant properties of BC under different pathological conditions [14,15,16], since under circumstances of strong oxidative stress, harmful derivatives are formed, aggravating the cellular and tissue damage [17,18,19,20]. Earlier, we investigated the effects of BC, administered at a low (30 mg/kg/day) and high dose (150 mg/kg/day), in intact and diabetic rat myocardium subjected to 30 min of ISA and 120 min of REP [15,16], since ISA and REP are associated with increased OS. In those studies, we found that BC treatment resulted in significant improvement of postischemic cardiac function, reduction of IS and increase in tissue antioxidant capacity, but only when it was administered at a low dose (LD), whereas these effects were abolished when the rats were treated with HD-BC. Furthermore, HD-BC treatment significantly increased HO-1 expression in the cardiac tissue. Although elevated HO-1 activity reduces OS and protects tissues from oxidative insult, in this case, cardiac ISA/REP-induced injury along with HD-BC treatment markedly activated HO-1, which could result in the increased release of Fe^2+^ from heme as a consequence of the HO-1 activity. Furthermore, it was shown that iron has a key role in myocardial ISA/REP-induced damage [21,22,23]. The underlying mechanisms of iron “toxicity” in the myocardium involve the increased formation of free radicals and reactive oxygen species (ROS) via the Fenton reaction [10,24], as well as the recently identified iron-dependent cell death ferroptosis [21,25]. One of the main sources of iron for these mechanisms is the activity of HO-1. Earlier, HO-1 was characterized as an antioxidant enzyme, which has a crucial role in different OS-related pathologies in many organs [26]. However, increasing evidence shows that it could function as a pro-oxidant by enhancing ROS production [27] and ferroptosis [28]. Furthermore, Sy C. et al. [29] showed that Fe^2+^ enhances the formation of various BC cleavage products (CPs), which suggests that increased HO-1 activity in the presence of high BC levels potentially overwhelms the cytoprotective effects of HO-1 and exacerbates OS on the myocardial tissue.

If iron overload associated with elevated HO-1 expression contributes at least partly to the diminished cardiac protection of a high BC dosage, then the elimination of excess iron from the tissue could abolish the earlier observed loss of protection. Thus, in the present study, we investigated the effects of DFO, an IC, which chelates the Fe^2+^ that participates in the Fenton reaction, thus preventing the formation of hydroxyl radicals and subsequent oxidative stress [25] on postischemic cardiac function, IS and HO-1 expression in isolated rat hearts subjected to 30 min of ISA followed by 120 min of REP after long-term (4 weeks) treatment with vehicle or HD-BC (150 mg/kg/day).

## 2. Results

### 2.1. Cardiac Function in ISA/REP-Injured Hearts Isolated from Vehicle- or BC-Treated Rats with or without DFO Treatment

**Figure 1:**
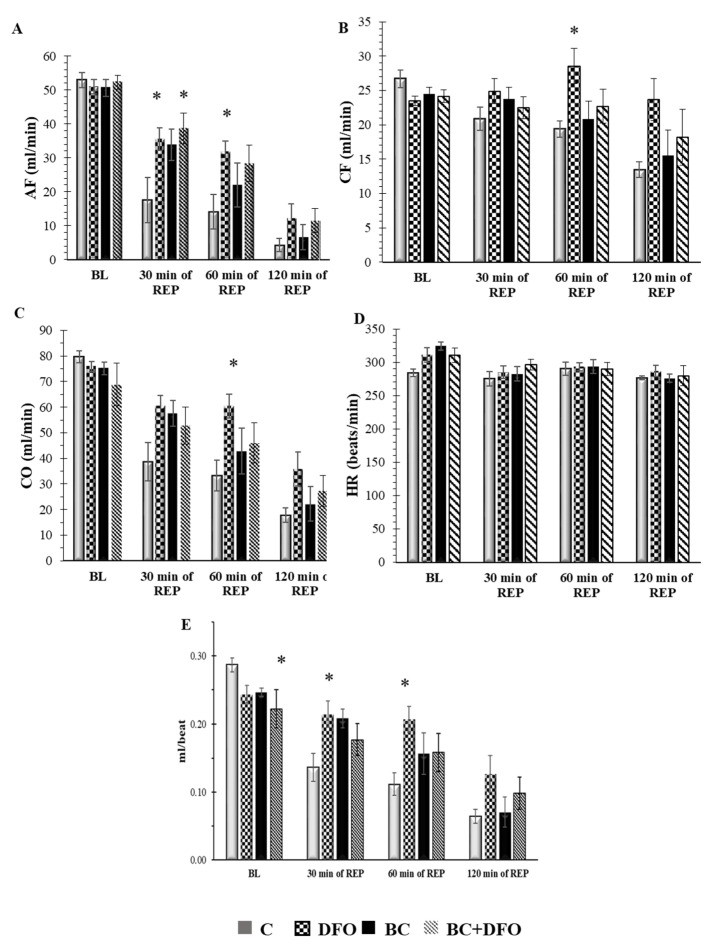
Effects of BC treatment and DFO administration on cardiac function in isolated working rat hearts. Hearts were subjected to 30 min of ISA followed by 120 min of REP in an isolated “working-heart” setup. Results are shown as mean ± SEM of AF (**A**); CF (**B**); CO (**C**); HR (**D**) and SV (**E**). (*n* = 7–9) * *p* < 0.05 in comparison with the magnitude of each cardiac function measured in each test group relative to hearts from vehicle-treated control rats. C: vehicle-treated control group; DFO: vehicle-treated, DFO-administered group; BC: BC-treated group without DFO administration; BC + DFO: BC-treated group with DFO administration. BL: base line; REP: reperfusion; AF: aortic flow; CF: coronary flow; CO: cardiac output; HR: heart rate; SV: stroke volume.

Figure 1 shows the effect of 0 or 150 mg/kg/day BC with or without DFO on cardiac function in hearts subjected to 30 min of ISA followed by 120 min of REP. Neither BC nor DFO-mediated effects were observed on the aortic flow (AF) of the hearts before the induction of ISA and REP (BL). In comparison with hearts from vehicle-treated control animals, DFO treatment resulted in a significant increase in AF after 30 and 60 min of REP (* *p* < 0.05), as well as in the case of BC-treated and DFO-administered hearts after 30 min of REP (* *p* < 0.05), but not in the case of hearts isolated from rats, which received BC treatment alone (Figure 1A). We did not observe any differences in the coronary flow (CF) in hearts before ISA (BL) or after 30 min of ISA followed by 30 or 120 min of REP; however, DFO treatment resulted in a significant increase in the CF of the hearts that originated from non-BC-treated rats after 60 min of REP compared with the vehicle-treated ones (* *p* < 0.05) (Figure 1B). An evaluation of the effects of DFO on cardiac output (CO) (Figure 1C) revealed no differences between the vehicle-treated control and test groups before the induction of ISA (BL) or 30 and 120 min of REP, whereas, after 60 min of REP, DFO significantly improved CO in comparison with the vehicle-treated hearts (* *p* < 0.05), but no differences were detected in the case of hearts isolated from BC-treated animals (Figure 1C). Treatment of the rats with BC and the administration of DFO had no effect on heart rate (HR) (Figure 1D). Likewise, for stroke volume (SV), there was a significant difference between the control- and BC-treated and the DFO-administered hearts (BC + DFO) before the induction of ISA (BL). No significant changes were observed after ISA/REP in BC-treated hearts; however, DFO administration significantly increased the SV of hearts isolated from vehicle-treated rats after 30 and 60 min of REP (* *p* < 0.05) (Figure 1E).

### 2.2. The Effect of BC Treatment and DFO Administration on ISA/REP-Induced IS

**Figure 2:**
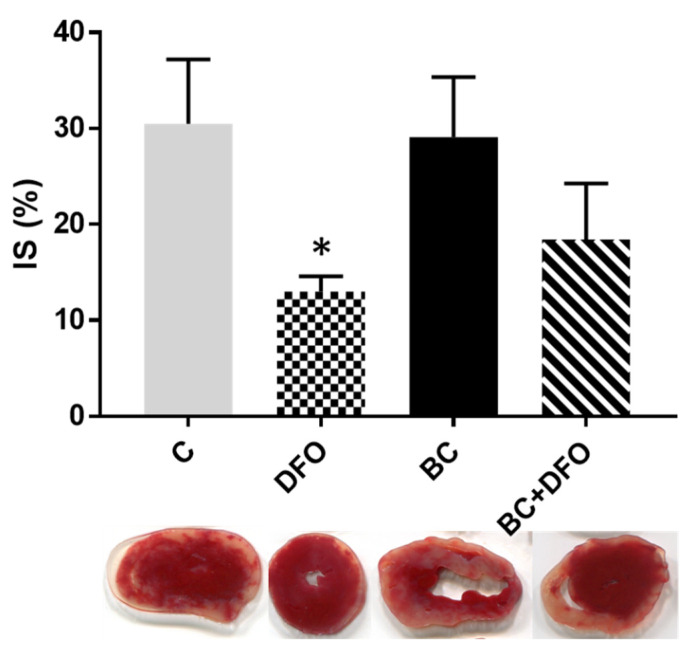
Effect of DFO administration on infarcted area in hearts isolated from vehicle- or BC-treated rats. Infarct size was measured in hearts (*n* = 4 in each group) after 120 min of REP by perfusing the hearts with triphenyl-tetrazolium-chloride (TTC) solution, followed by macroscopic analysis of transverse sections of each heart. Data are expressed as average size (%) of infarcted area ± SEM for each group. * *p* < 0.05 in comparison with the values for hearts from vehicle-treated control rats. Representative pictures are also shown under the bars. White areas indicate infarcted tissues using triphenyltetrazolium chloride staining, whereas red color represents the survival regions. C: vehicle-treated control group; DFO: vehicle-treated, DFO-administered group; BC: BC-treated group without DFO administration; BC + DFO: BC-treated group with DFO administration.

The extent of the effect of DFO treatment on the infarcted area in ISA/REP-injured hearts is shown in Figure 2. Macroscopic analysis of the heart sections revealed that DFO administration resulted in a significant reduction in the extent of infarcted myocardium relative to the control hearts isolated from vehicle-treated rats (* *p* < 0.05) (Figure 2-DFO). This protective effect cannot be seen in hearts isolated from BC-treated animals without DFO administration (Figure 2-BC). It was further noted that the IS in the hearts of rats treated with HD-BC (150 mg/kg/day) was reduced after the administration of DFO (Figure 2-BC + DFO); however, it was not reduced at a significant level.

### 2.3. HO-1 Protein Expression in Hearts Isolated from Vehicle- or BC-Treated Rats with or without the Administration of DFO

**Figure 3:**
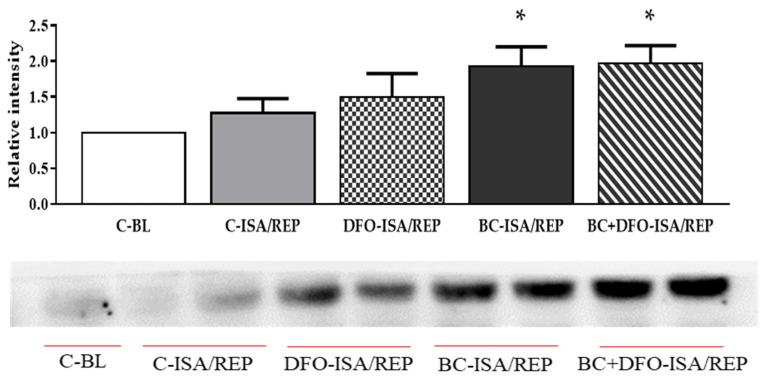
Western blot analysis of HO-1 protein level in cardiac tissue. Expression of HO-1 protein in the hearts was measured in homogenized myocardial tissue samples from vehicle- or BC-treated rats with or without DFO treatment and ISA/REP injury. Tissue content of HO-1 is shown as a ratio of arbitrary units for HO-1 protein to total protein. Data are expressed as mean ± SEM of 7 different blots. * *p* < 0.05 compared to non-ischemic control (C-BL) hearts. A representative blot is also shown under the bars. Original blots are provided as supplementary Figure S1. C-BL: non-ischemic control group; C-ISA/REP: vehicle-treated, ischemic/reperfused control group; DFO-ISA/REP: vehicle-treated, ischemic/reperfused group with DFO administration; BC-ISA/REP: BC-treated, ischemic/reperfused group; BC + DFO-ISA/REP: BC-treated, ischemic/reperfused group with DFO administration.

The HO-1 expression of cardiac tissue was measured by Western blot analysis (Figure 3). The results show that, in control hearts, which were not subjected to ISA/REP (C-BL), HO-1 expression was not significantly different from ISA/REP-injured hearts with or without DFO treatment (C-ISA/REP and DFO-ISA/REP) excised from vehicle-treated rats (Figure 3). Furthermore, the production of HO-1 protein in ISA/REP-injured hearts from rats that received BC treatment was significantly higher compared to the non-treated, non-injured (C-BL) group (* *p* < 0.05) irrespective of the administration of DFO (Figure 3-BC-ISA/REP and BC + DFO-ISA/REP).

## 3. Discussion

Increased OS is considered one of the major factors in the development and/or progression of many chronic diseases, including diabetes, cancer, neurodegenerative and cardiovascular disorders, as well as aging [30]. During OS, ROS and reactive nitrogen species (RNS) are produced in excess, attacking and oxidizing biological macromolecules, such as DNA, membrane lipids and proteins, finally leading to cell death. Thus, the effective modulation of OS by the development of new antioxidant therapies is crucial for the survival of the cells and for the prevention of the development as well as the progression of OS-related diseases [1]. It is reasonable to assume that the progression and/or development of these disorders could be prevented or at least delayed by the administration of different dietary antioxidants, including BC, since one of the major contributors to OS is the free radicals ROS and RNS. Although BC was characterized as an antioxidant molecule, increasing evidence suggests that it possesses pro-oxidant characteristics or loses its antioxidant effect depending on the circumstances and the applied concentration [31,32]. Moreover, it was shown that BC loses its protective effects against oxidative injury at high doses [33,34]. In our earlier studies, we observed the same effects of BC [15,16]. In those experiments, we studied the effects of the molecule under ISA/REP-induced increased oxidative circumstances in rat hearts isolated from healthy and Zucker diabetic fatty (ZDF) rats after long-term, low- and high-dose BC treatments. We found that BC improved cardiac function and decreased IS only when it was administered at an LD, and this cardioprotective effect was lost when BC was administered at a high concentration in spite of an elevated HO-1 level [15,16], and this observation was also seen in the present study (Figure 1, Figure 2 and Figure 3) in the case of the BC group. The loss of the protective effect may be related to the increased formation of different carotenyl radicals and BC CPs under the strong oxidative circumstances of ISA/REP. It was proposed that these CPs resulted in mitochondrial dysfunction and increased O_2_^−^. production as well as the accumulation of H_2_O_2_ [19]. It is supported, however indirectly, by the findings of Da Rocha et al. [35], who observed the increased oxidation of lipids and proteins, decreased catalase (CAT) activity, SOD/CAT ratio and reduced mitochondrial respiratory chain complex activities in rat hearts after long-term HD vitamin A treatment. As a consequence of the decreased CAT activity, H_2_O_2_ accumulates in the tissue, and in the presence of transition metals, especially Fe^2+^, induces the Fenton reaction, producing hydroxyl radicals in excess, which further enhance cellular and tissue damage. Previously [15,16], as well as in this study, we measured an increased HO-1 level in ISA/REP-injured hearts isolated from BC-treated rats (Figure 3-BC and BC + DFO), which supports the observed loss of cardiac protection since Fe^2+^ was also produced by HO-1 activity. Furthermore, it had been shown that iron induces the auto-oxidation of BC and increases its pro-oxidant activity [29,36]. Moreover, iron takes part in the pathogenesis of ISA/REP as well as in ferroptosis [21,37,38]. Thus, the removal of free iron from the tissue could be essential to the reduction of ISA/REP-induced cardiac injuries; hence, the administration of ICs should exert protective effects. In the present study, we applied DFO in order to eliminate excess iron and reduce the ISA/REP-induced cardiac damage. The results show that DFO alone improved cardiac function (Figure 1-DFO) and decreased IS (Figure 2-DFO), similarly to the result of [38], in hearts isolated from non-BC-treated rats subjected to 30 min ISA followed by 120 min REP. This observation is in good correlation with the results of Gao M. et al. [39], who found that the administration of DFO prevented ferroptosis, reduced IS and improved cardiac function in isolated, ischemic/reperfused mice hearts. Furthermore, in their study, Tang et al. [38] investigated the effect of DFO on ferroptosis in ischemic/reperfused rat hearts. The authors found that the administration of DFO significantly decreased IS and reduced OS parallel with the reduction in the iron level of the hearts [38]. Since, in our earlier studies, we found that BC treatment along with ISA/REP injury resulted in an elevated HO-1 level [15,16], we also tested the effects of DFO in hearts isolated from BC-treated rats (BC + DFO). Contrary to our initial expectations, the application of DFO did not affect or only moderately affected cardiac function (Figure 1) and the IS (Figure 2). Although we observed a mild improvement, this was not statistically significant (Figure 1 and Figure 2-BC + DFO). An explanation of this may involve the high level of oxidative CPs formed from BC under strong oxidative circumstances [40], since ROS and RNS are produced in excess during ISA/REP. This is supported by the fact that we measured a significantly lower tissue antioxidant capacity in the hearts after HD-BC treatment [15]. Elevated levels of these kinds of compounds together with high free-iron content may accelerate OS-induced cardiac damage via the Fenton reaction, initiating a *circulus vitiosus*, and maybe DFO at the applied concentration is not able to completely remove the excess Fe^2+^ and diminish the negative effects of iron release originating from the high BC dose related to increased HO-1 activity. The potential negative effects of increased HO-1 expression and activity in connection with an excess iron level were also studied by other research groups under different pathological circumstances [41,42,43]. It was shown by Farhangkhoee H. et al. [41] that increased HO-1 expression and activity under enhanced oxidative circumstances induce the accumulation of iron in the cardiac tissue. Furthermore, they observed that the induction of HO-1 was also associated with increased OS. In another study, Khan Z. A. et al. [42] found that the induction of HO-1 led to iron accumulation parallel with increased oxidative stress in the liver. The inhibition of the enzyme reversed the observed effects. Earlier, Suttner and Dennery [43] showed that high HO-1 expression and activity are associated with an increased reactive iron level and enhanced oxidative stress in HA-1 cells subjected to hyperoxia, and this oxygen toxicity is decreased by the inhibition of HO-1 or the chelation of the iron before the oxidative injury. On the other hand, the concentration of DFO we used could be enough to eliminate the iron released during ISA/REP insult in hearts without BC treatment since, in this case, we observed lower HO-1 expression compared to BC-treated hearts; consequently, the iron load of the cardiac tissue was lower, which was reflected in improved cardiac function (Figure 1-DFO) and decreased IS (Figure 2-DFO). Furthermore, based on our results, we may speculate that free iron is one component that abolishes the protective effects of BC applied at a high concentration since chelation of free iron only partially restored cardiac protection, which we observed in the case of LD-BC treatment [15,16]. In order to completely reverse the lost protection, DFO should be applied in higher concentrations, or other molecules should be tested, which may be more potent than DFO. A suitable candidate could be N-acetyl-L-carnosine (NAC), a dipeptide, which is the acetylated form of L-carnosine and can be found in different organs of mammals, including the heart. It has been shown that NAC possesses good antioxidant activity and is able to reduce the malondialdehyde level originating from the iron-induced oxidation of liposomes in an Fe^2+^-ascorbate system [44,45]. Furthermore, it has good metal-chelating activity as well [45]. NAC can be considered the prodrug of L-carnosine, which has well-documented antioxidant activities under different pathological conditions [46]. The advantage of NAC against its parent drug is that it is more soluble in lipids; consequently, it may pass through the cell as well as mitochondrial membranes more easily [45]. Moreover, Alabovsky V. V. et al. [47] investigated the effects of the molecule in isolated rat hearts subjected to ischemia and reperfusion. The authors found that NAC improved the contractility of the hearts and protected the membranes from ischemia/reperfusion-induced damages. It has to be mentioned that the concentration of BC (150 mg/kg) we used was very high—higher than the therapeutic values. However, it is important to note that the doses applied in humans are 70–100 times less compared to the doses used in animal experiments. Since the absorption of carotenes from the gastrointestinal system of rats is very low, it was necessary to administer BC at higher doses. A detailed explanation of the dose selection was critically discussed in our earlier article [15]. Furthermore, it has to be mentioned that the limitation of the current study originates from the used experimental model since the translational value of the results obtained using rats treated with carotenes is limited. However, these animals are frequently used in cardiovascular research, but this was discussed in detail in our previous publication [16].

## 4. Materials and Methods

The experiments were accomplished using adult male Sprague-Dawley rats (Charles River Laboratories) with a body weight range of 350–400 g. The animals were housed in wire-bottomed cages (three rats per cage) throughout the study (4 weeks) and provided with normal laboratory rodent chow pellets (R/M-Z + H, ssniff Spezialdiäten GmbH, Soest, Germany) and water *ad libitum* and kept at an ambient temperature of 25 ± 2 °C with a relative humidity of 55 ± 5% and a 12-h light-dark cycle. All rats received humane care in compliance with the “Principles of Laboratory Animal Care” (formulated by the U.S. National Society for Medical Research, as described in U.S. National Institutes of Health, publication No. 86-23, revised 1996) and the “Guide for the Care and Use of Laboratory Animals”. Maintenance and treatment of rats used in the present study were additionally approved by the Institutional Animal Care and Use Committee of the University of Debrecen, Debrecen, Hungary (6/2019/DEMAB).

### 4.1. Treatment Protocol and Isolated Heart Preparation

Rats were segregated into 4 groups (*n* = 10 in each group). Two groups received hydroxyethyl cellulose-water (1:4) while animals in the remaining two groups were treated with BC (150 mg/kg/day) suspended in hydroxyethyl cellulose-water (1:4) by gavage daily for 4 weeks, respectively. All-trans-BC was obtained from Merck Kft. (Budapest, Hungary). Following the 4-week treatment with vehicle or BC, the rats were anesthetized with an intraperitoneal ketamine-xylazine injection (75/10 mg/kg), and intravenous heparin was given as anticoagulant (1000 IU/kg). Following the induction of deep anesthesia, chest cavities were opened, and hearts were excised and placed in ice-cold modified Krebs-Henseleit bicarbonate (KHB) buffer to prevent damage to cardiac tissue. After excision of the hearts, aortas were cannulated, and hearts were perfused with modified KHB buffer at a filling pressure of 100 cm of water using the Langendorff (non-working) mode of the isolated working heart setup for 5 min to flush blood out from the hearts. During the washout period, pulmonary veins were cannulated, and heart function was assessed in working mode at a filling pressure of 17 cm of water with KHB buffer [15].

Hearts obtained from rats receiving vehicle were further divided into two groups. The first group was used as control (C), and hearts in the other group were treated with DFO (100 μM) during the REP period of the ex vivo experiments. Similarly, hearts isolated from BC-treated rats were also further divided into two groups, and the first group was not treated with DFO (BC), while hearts in the other group received DFO (100 μM) treatment (BC + DFO) during REP of the isolated heart experiments. DFO was dissolved in the perfusion buffer.

### 4.2. Induction of ISA/REP and Cardiac Function Assessments

Following 10 min of aerobic working perfusion, 30 min of global ISA was initiated by clamping the pulmonary inflow and the aortic outflow. At the end of the ischemic period, 120 min of REP was started by unclamping the inflow and outflow lines. The first 10 min of REP was conducted in Langendorff mode to avoid the development of fatal ventricular arrhythmias. Baseline parameters for each heart were registered following the 10 min of working perfusion before the induction of ISA. To examine the recovery of the left ventricle, cardiac function was assessed after 30, 60 and 120 min of REP. During the entire experimental procedure, HR was measured by a computer acquisition system (ADInstruments, PowerLab, Castle Hill, Australia). AF was measured by a calibrated flow meter. CF was assessed by the timed collection of the effluent dripping from the heart. CO was calculated as a sum of AF and CF. SV was generated as the ratio of CO and HR [15].

### 4.3. Determination of Infarct Size

Estimations of infarcted area were conducted using the TTC staining method as previously described [15]. Briefly, following each 30-min ISA/120-min REP period, hearts were perfused with 50 mL 1% (*w*/*v*) solution of TTC in phosphate buffer (pH 7.4), and the samples were stored at −70 °C for subsequent analysis. The frozen samples were sectioned, weighted, and blotted dry. The dried sections were scanned on an Epson J232D flat-bed scanner (Seiko Epson Corporation, Nagano, Japan). The infarcted area (white coloration) and the risk area (entire scanned section) were measured using planimetry software (Image J, National Institute of Health, Bethesda, MD, USA). Estimates of infarcted zone magnitude were subsequently obtained by multiplying infarcted areas by weight of each slice. The resulting numbers represent weight of the risk zone and the infarcted zone. IS was expressed as percentage of the weight of infarcted tissue and the weight of risk zone (whole heart).

### 4.4. Protein Isolation and Western Blot Analysis

Approximately 300 mg of cardiac tissues was homogenized using a Polytron homogenizer in isolating buffer (25 mM Tris-HCl, 25 mM NaCl, 1 mM orthovanadate, 10 mM NaF, 10 mM pyrophosphate, 10 mM okadaic acid, 0.5 mM EDTA, 1 mM PMSF and 1× protease inhibitor cocktail) and centrifuged at 358× *g* at 4 °C for 10 min. The supernatant was transferred to a new tube and centrifuged at 8944× *g* at 4 °C for 20 min, and then the supernatant was used as a cytosolic fraction. Protein concentration was measured by a BCA Protein Assay Kit (Thermo Scientific, Rockford, IL, USA).

A total of 50 μg of protein in each sample was separated on TGX Stain-Free™ 12% acrylamide gels. Then, gels were exposed to UV light; thereby, trihalo compounds contained in stain-free gels covalently bind to tryptophan residues in proteins, allowing total protein quantification. After transferring the proteins to PVDF membranes for 1 h at 100 V, membranes were exposed to another brief irradiation, and the resulting fluorescence signals were recorded, and the signal intensity is proportional to the total protein volume. After blocking with 5% of non-fat dry milk in TBST, membranes were incubated with primary antibody solution at 4 °C overnight. HO-1 antibody was obtained from Abcam (Cambridge, UK), 1:1000. The membranes were washed with TBST and incubated with HRP-conjugated secondary antibody solution. After washing, the membranes were incubated with Clarity Western ECL substrate (Bio-Rad Laboratories, Hercules, CA, USA) to visualize through enhanced chemiluminescence bands according to the recommended procedure (ChemiDoc Touch, Bio-Rad Laboratories, Hercules, CA, USA). The chemiluminescent bands and each total protein lane intensity were measured by Image Lab 5.2.1 software (Bio-Rad Laboratories, Hercules, CA, USA). During quantification, protein density was measured directly on the membranes and reflected to total loaded proteins. Thus, this type of normalization eliminates the need to select housekeeping protein [48,49].

### 4.5. Statistical Analysis

Statistical analyses were performed using GraphPad Prism 7 software (GraphPad Software Inc., La Jolla, CA, USA). The data are expressed as mean +/− SEM. One-way ANOVA followed by Dunnett’s post-hoc test was carried out for data analysis. Differences were considered to be significant at the value of *p* < 0.05.

## 5. Conclusions

In conclusion, our results suggest that iron produced by increased HO-1 activity in response to ISA/REP after long-term HD-BC treatment could have an important role in ISA/REP-induced cardiac injury. DFO, a hydrophilic IC, had only a mild effect on cardiac function and IS at the applied concentration in the case of hearts isolated from BC-treated rats; thus, higher dose(s) of DFO should be applied. Furthermore, additional experiments are necessary to clarify the precise role of HO-1 and iron in mediating the cardiovascular effects of BC. The modulation of the effects of iron related to HO-1 activity should be investigated by the application of other types of synthetic or natural iron chelators (e.g., NAC) and/or via the modulation of the keap1/Nrf2/HO-1 pathway by the application of the inhibitors of these signaling molecules. Furthermore, the inhibition of the ferroptotic signaling pathways could also be a good target for the modulation of the effects of iron originating from increased HO-1 activity as a consequence of HD-BC treatment. The precise role of BC under ischemia and reperfusion in connection with iron needs to be further elucidated.

## Data Availability

The data presented in this study are available on request from the corresponding author.

## Supplementary Materials

The following supporting information can be downloaded at: https://www.mdpi.com/article/10.3390/molecules27093039/s1, Figure S1: Original Western blots. Western blot analysis of HO-1 protein expression level in cardiac tissue. During quantification, protein density was measured directly on the membranes and reflected to total loaded proteins. Each total protein lane intensity (upper membranes) and chemiluminescent bands (lower membranes) were measured by Image Lab 5.2.1 software. The red arrows show the chemiluminescent bands, which were evaluated. The samples were loaded into the numbered wells in the following order on each membrane: protein ladder; then 1: C-BL; 2–3: C-ISA/REP; 4–5: DFO-ISA/REP; 6–7: BC-ISA/REP; 8–9: BC + DFO-ISA/REP. C-BL: non-ischemic control group; C-ISA/REP: vehicle-treated, ischemic/reperfused control group; DFO-ISA/REP: vehicle-treated, ischemic/reperfused group with DFO administration; BC-ISA/REP: BC-treated, ischemic/reperfused group; BC + DFO-ISA/REP: BC-treated, ischemic/reperfused group with DFO administration.

## Sample Availability

Samples of the compounds are not available from the authors.
