# Supplementary material for: Beta-Carotene Affects the Effects of Heme Oxygenase-1 in Isolated, Ischemic/Reperfused Rat Hearts: Potential Role of the Iron
Source: Molecules. 2022 May 9;27(9):3039. doi: 10.3390/molecules27093039 (PMC9101800; doi:10.3390/molecules27093039)
Supplement: Supplementary file 1 [file molecules-27-03039-s001.zip › molecules-1634463-supplementary.pdf]

Supplementary

Figure S1: Original Western blots.

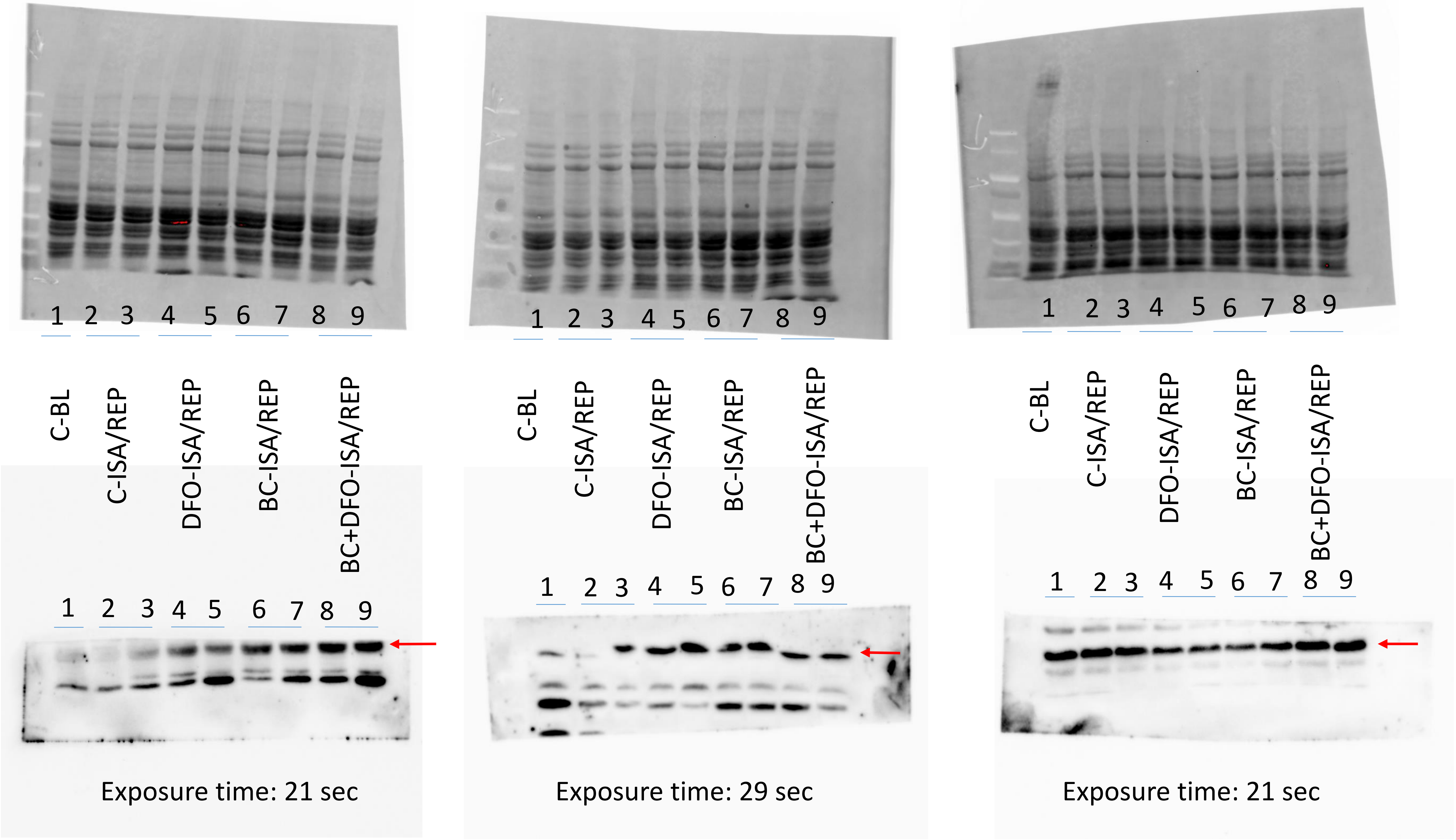
1 2 3 4 5 6 7 8 9
C-BL
C-ISA/REP
DFO-ISA/REP
BC-ISA/REP
BC+DFO-ISA/REP
Exposure time: 21 sec
Exposure time: 29 sec
Exposure time: 21 sec

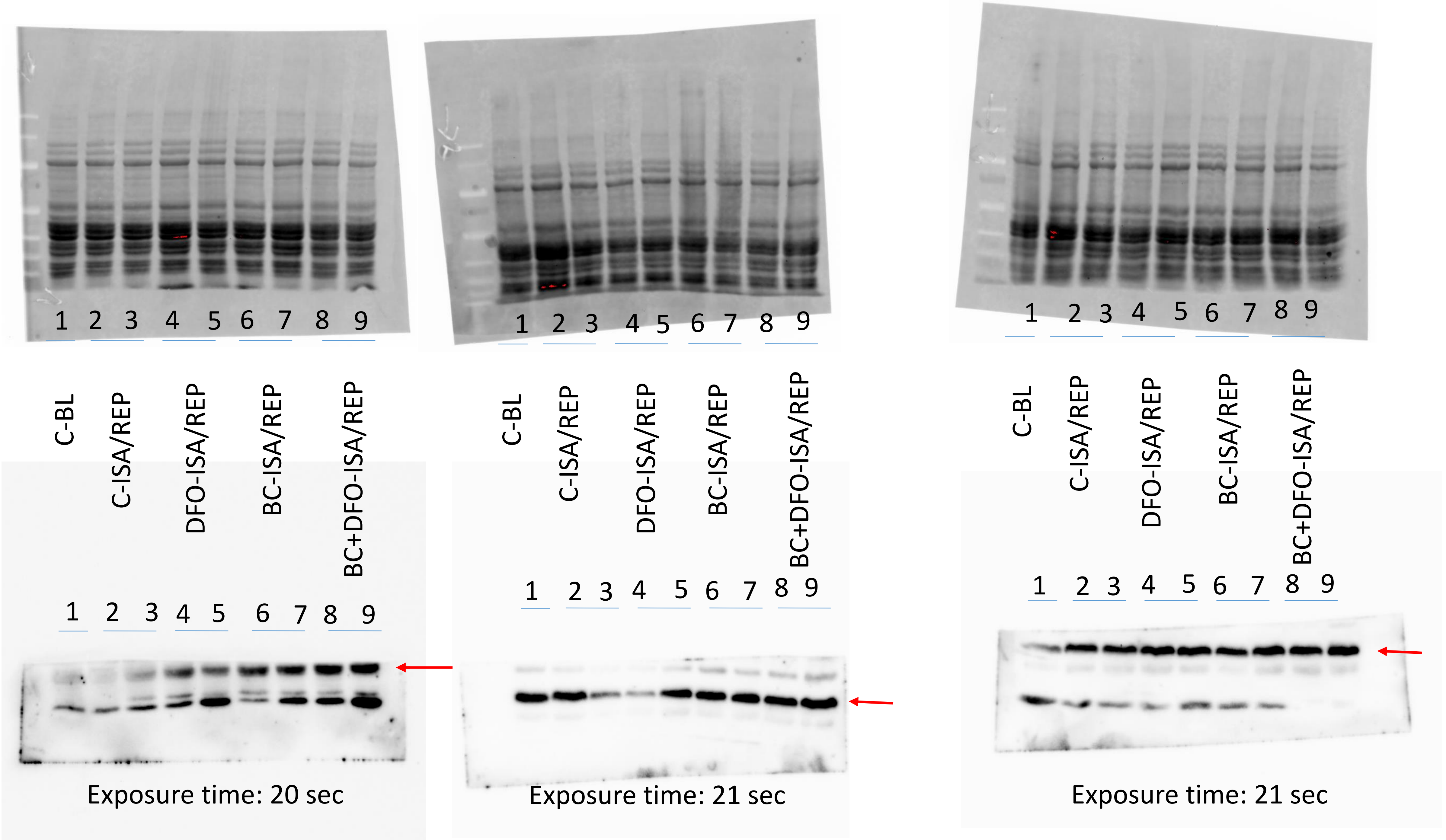
1 2 3 4 5 6 7 8 9
C-BL
C-ISA/REP
DFO-ISA/REP
BC-ISA/REP
BC+DFO-ISA/REP
Exposure time: 20 sec
C-BL
C-ISA/REP
DFO-ISA/REP
BC-ISA/REP
BC+DFO-ISA/REP
Exposure time: 21 sec
C-BL
C-ISA/REP
DFO-ISA/REP
BC-ISA/REP
BC+DFO-ISA/REP
Exposure time: 21 sec

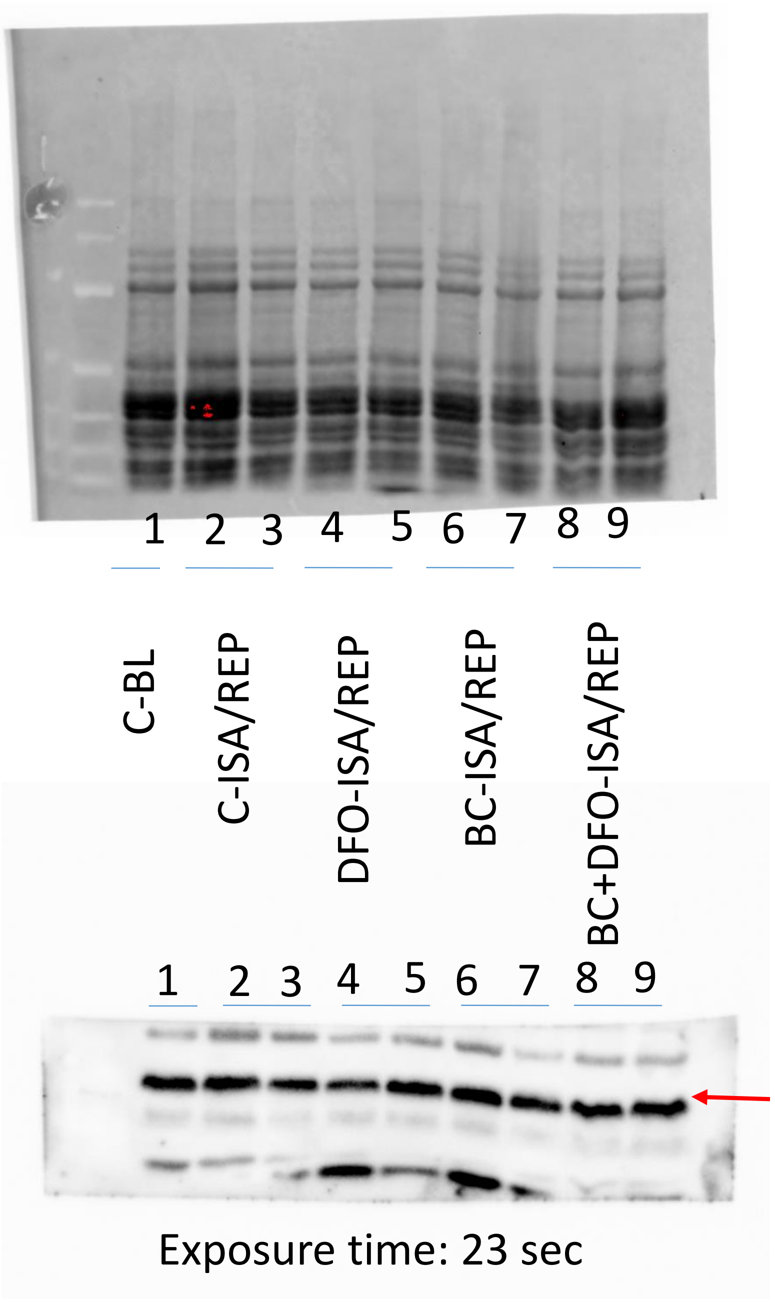
1 2 3 4 5 6 7 8 9
C-BL
C-ISA/REP
DFO-ISA/REP
BC-ISA/REP
BC+DFO-ISA/REP
1 2 3 4 5 6 7 8 9
Exposure time: 23 sec
